# Endogenous Phenolics in Hulls and Cotyledons of Mustard and Canola: A Comparative Study on Its Sinapates and Antioxidant Capacity

**DOI:** 10.3390/antiox3030544

**Published:** 2014-08-15

**Authors:** Shyamchand Mayengbam, Ayyappan Aachary, Usha Thiyam-Holländer

**Affiliations:** 1 Department of Human Nutritional Sciences, University of Manitoba, Winnipeg, MB R3T 2N2, Canada; E-Mails: Ummayens@cc.umanitoba.ca (S.M.); Ayyappan.Appukuttan@ad.umanitoba.ca (A.A.); 2 Richardson Centre for Functional Foods and Nutraceuticals & Department of Human Nutritional Sciences, University of Manitoba, Winnipeg, MB R3T 2N2, Canada

**Keywords:** sinapic acid derivatives, sinapine, sinapoyl glucose, mustard, canola, hulls, cotyledons, antioxidant activity

## Abstract

Endogenous sinapic acid (SA), sinapine (SP), sinapoyl glucose (SG) and canolol (CAN) of canola and mustard seeds are the potent antioxidants in various lipid-containing systems. The study investigated these phenolic antioxidants using different fractions of canola and mustard seeds. Phenolic compounds were extracted from whole seeds and their fractions: hulls and cotyledons, using 70% methanol by the ultrasonication method and quantified using HPLC-DAD. The major phenolics from both hulls and cotyledons extracts were SP, with small amounts of SG, and SA with a significant difference of phenolic contents between the two seed fractions. Cotyledons showed relatively high content of SP, SA, SG and total phenolics in comparison to hulls (*p* < 0.001). The concentration of SP in different fractions ranged from 1.15 ± 0.07 to 12.20 ± 1.16 mg/g and followed a decreasing trend- canola cotyledons > mustard cotyledons > mustard seeds > canola seeds > mustard hulls > canola hulls. UPLC-tandem Mass Spectrometry confirmed the presence of sinapates and its fragmentation in these extracts. Further, a high degree of correlation (*r =* 0.93) was noted between DPPH scavenging activity and total phenolic content.

## 1. Introduction

Over the last two decades, the positive effects of endogenous bioactive phenolic compounds from plants and oilseeds have received considerable attention due to the role of phenolic antioxidants in human nutrition and health [1,2]. Rapeseed (*Brassica napus* L. spp. *oleifera*), an excellent source of phenolic antioxidants, is one among the 100 species in the *Brassica* *genus*. Additionally, canola that is another variety of *B*.* napus* contains various minor constituents such as tocopherols, carotenoids, phytic acid, sinapic acid (SA), and its derivatives (SADs) namely sinapine (SP), the choline ester of SA and sinapoyl glucose (SG), the glucose ester of SA and low levels of erucic acid (~2%) and glucosinolates (<30 μg/g). The relatively high concentration of both free and esterified SA was reported in press cakes and proteins of rapeseed [3]. The SA and the decarboxylation product canolol (CAN) are considered to be potent antioxidants as demonstrated recently by various *in vitro* assays in various food products. The endogenous bioactive principles of canola and rapeseeds have great potential as therapeutic agents to maintain and improve human health and well-being, and can be incorporated into many food and non-food products. There are, however, several questions that still need to be answered with respect to SADs distribution in various fractions of canola and mustard seeds to be able to understand their antioxidant efficacy in bulk oil and emulsion systems.

Oilseeds are potential sources for various bioactive molecules such as phenolics and glucosinolates and most of them are retained in oilseed processing by-products (meal, press cakes, and hulls) in significant amounts. The isolation of these bioactive molecules is justified in the value addition perspective of these by-products. With respect to various factors, especially the genetics of the rapeseed, and the processes of oil extraction, the contents of SADs in rapeseed meal vary significantly (6–18 mg/g) [4,5]. Among various SADs of rapeseed meal, the glucose ester of SA (SG) is highly potent in terms of its antioxidant efficacy [6]. SA is a potential peroxyl radical scavenger [7], and it had been demonstrated to retard oxidation process in many emulsion systems including bulk methyl linoleate (MeLo), emulsified MeLo, sunflower oil methyl esters and low-density lipoprotein [7,8,9,10]. Wanasundara *et al*. proved that, for the oxidation of liposomes and low-density lipid particles, SP is the principal contributor towards the antioxidative potential of phenolic extracts from rapeseed [11]. Various phenolic bioactive constituents of rapeseed meal and crude oil were also shown to have antioxidative properties [4,6,12,13]. A significant reduction (>90%) in the oxidation of LDL particles by rapeseed phenolics was reported [14]. The antioxidant effectiveness of canola hulls extracts in methanol and acetone was comparable to butylated hydroxyanisole in model systems of β-carotene-linoleate [15]. The rapeseed phenolics were better antioxidants towards liposomes membrane oxidation and the radical scavenging activities of such phenolics from rapeseed oil were significantly high [16]. The authors also suggested the possible use of rapeseed phenolics in functional food product development, owing to its abundance and potent bioactive attributes.

Wakamatsu *et al*. isolated 4-Vinylsyringol or CAN from crude canola oil [17] and it was reported that the decarboxylation of SA via roasting treatments could increase CAN content of rapeseed [18]. CAN is a highly effective 1,1-diphenyl-2-picrylhydrazyl (DPPH) radical scavenger [13,14,19] and it inhibits oxidative degradation of lipids and proteins [16]. Kuwahara *et al*. studied scavenging capacity of CAN with respect to peroxynitrite, which is an endogenous mutagen and reported that CAN effectively suppressed peroxynitrite-induced bactericidal action [20].

Recently Bala and Singh developed a Near Infra-Red spectroscopy method allowing rapid and non-destructive detection of total phenolics in mustard using whole seed [21]. However, high-performance liquid chromatography-diode array detector (HPLC-DAD) is proven to be the most convenient, efficient, and reliable technique to quantify phenolics [22,23,24,25,26]. The quantitative profile of SADs in commercial canola and mustard products and their extracts need to be established. There is a lack of information on the concentration of phenolic antioxidants of commercial oil seeds. Recently, Yang *et al*. estimated the concentration of some of the minor components of rapeseed oil produced via cold-pressing of a few varieties cultivated in Yangtze River Valley, China [27]. However, the study was not extensively carried out on the phenolic constituents but on the total phenolic concentration which was reported to be 36 mg/100 g of sample.

The phenolic compounds non-uniformly distributed in different fractions of oilseed, where in certain fractions have more phenolics than others. However, there are no data on the *in-situ* distribution of phenolics in different sections of an oilseed, particularly canola or mustard. This information will further strengthen the rationale of value addition of oilseed by-products such as hulls, meals, and press cakes for recovering phenolics. In this scenario, the purposes of our study were to identify, quantify, and characterize the antioxidant phenolics of hulls and cotyledons of canola and mustard seeds. The study encompassed the identification and quantification of SADs by reversed-phase HPLC–DAD at 330 and 275 nm to understand the distribution of these phenolics between hulls and cotyledons. The study also used the quantitative comparison of total phenolic contents following two methods: HPLC and Folin-Ciocalteu’s method to ascertain the applicability of these methods in canola/rapeseed phenolics. Additionally, the major phenolics were characterized using ultra-high performance liquid chromatography-mass spectrometry (UPLC-MS), and the potential DPPH scavenging of these various residues of canola and mustard seeds was also investigated. This knowledge is essential to contribute to the optimized extraction of canolol from sinapic acid and other precursors.

## 2. Experimental Section

### 2.1. Chemicals and Materials

Analytical grade chemicals were used. Standards of sinapic acid and sinapine were procured from Sigma-Aldrich (St. Louis, MO, USA) and EPL Bioanalytical Services (Niantic, IL, USA) respectively. Dr. A. Baumert kindly donated standard of sinapoyl glucose. Sinapinaldehyde was a product of ChromaDex Inc. (Irvine, CA, USA). An authentic standard of 4-Vinylsyringol (canolol) was kindly donated by Amy Logan of CSIRO Animal Food and Health Sciences, Werribee, Australia. Mustard seeds were procured from G.S. Dunn Limited, Ontario, Canada and a local store. Dow AgroSciences (Calgary, AB, Canada) supplied Nexera, a variety of canola.

### 2.2. Phenolic Extraction from Hulls and Cotyledons of Canola and Mustard

Hulls and cotyledon of canola and mustard were manually separated. Whole seeds, hulls and cotyledons were defatted with *n*-hexane using Soxtec 2050 and extracted as per Thiyam *et al.* 2004 [28]. Briefly, 1 g of defatted canola or mustard fractions were extracted thrice in aqueous methanol (70%) assisted by ultra-sonication (60 s) followed by refrigerated centrifugation at 5000 rpm for 10 min. The filtrates from the three extractions, obtained through the filtration of methanolic layers using Whatman No. 1 filter paper from Sigma-Aldrich (St. Louis, MO, USA), were combined and made up to a known volume (25 mL). All the extractions were conducted in triplicates.

### 2.3. Total Phenolic Content of Canola and Mustard Seed Fractions

Phenolic contents of different fractions of canola and mustard were characterized by reversed phase HPLC-DAD [29]. Folin-Ciocalteu’s reagent based assay was used to estimate the total phenolics [30] with slight modifications. Briefly, the extracts, were appropriately diluted (2.5 fold) with distilled water, and 500 μL of this was thoroughly mixed with Folin-Ciocalteau’s phenol reagent (1:1 ratio). After a specified reaction period (3 min), 1 mL of 19% Na_2_CO_3_ was added, followed by monitoring of absorption at 750 nm after 60 min in a DU 800 UV/Visible Spectrophotometer (Beckman Coulter Inc., Mississauga, ON, Canada). The analysis was carried out in duplicates and compared with a calibration graph of SA and the results were expressed as SA equivalents (SAE).

### 2.4. DPPH Scavenging Activity

Different fractions of canola and mustard were assessed for their DPPH radical scavenging activities following Schwarz *et al*. method with slight modifications [31]. In covered test tubes (three for each sample), 100 μL of the phenolic extracts were combined with methanolic DPPH (2.9 mL, 0.1 mM). The tubes were vortexed thoroughly and placed in a dark cabinet for exactly 10 min before measuring the absorption values at 516 nm. The absorbance of control (A_c_) and absorbance of the sample (A_s_) were used to calculate scavenging effect (%), which is the percentage change in absorbance (A_c_–A_s_) with respect to A_c_. To calculate the EC_50_ concentration, different concentrations of 100 μL sample were used (20, 40, 60, 80, 100 μL of the sample and all of them were made to 100 μL using 80, 60, 40, 20 & 0 μL of methanol).

### 2.5. HPLC-DAD Analysis of Phenolic Compounds in Canola and Mustard Extracts

The phenolic profile of canola and mustard extracts was established following a reversed-phase HPLC-DAD (Ultimate 3000; Dionex, Sunnyvale, CA, USA) analysis [29]. Solvent A, 90% methanol (aqueous) acidified with *o*-phosphoric acid (1.2%) and solvent B, 100% methanol acidified with o*-*phosphoric acid (0.1%) were used as mobile phases in a gradient elution, where in the concentration of mobile phase B (%, indicated in brackets) changed in the following sequences at specified time periods (min) 0 (10), 7 (20), 20 (45), 25 (70), 28 (100), 31 (100) and 40 (10). Synergi 4 μ Fusion-RP 80 Å; 150 × 4.0 mm- 4 micron (Phenomenex, Torrance, CA, USA) column was used for SADs separation. Both the mobile phases and canola and mustard extracts were passed through syringe filters (0.45 μm). The following conditions of analysis were maintained: flow rate (1 mL/min), column compartment temperature (25 °C) and wavelengths of analysis (275 nm and 330 nm). Version 6.8 of Chromeleon software (Dionex Corporation, Sunnyvale, CA, USA) was used to acquire the HPLC data. Standards of SP, SG, SA, and CAN were also analyzed for comparison purpose based on retention time. Triplicate samples were analyzed for statistical validation of results.

### 2.6. UPLC-MS Analysis of Phenolics from Hulls and Cotyledons

A Waters Acquity UPLC system coupled to a Quattro micro API tandem mass spectrometer (Milford, MA, US) was used for the confirmation of SA and SADs. A Phenomenex Synergi 4 μ Fusion-RP column (150 × 4 mm, Torrance, CA, USA) was employed for the separation of SADs. 5 mM of ammonium acetate (pH 3.2 with acetic acid) and 100% methanol were the two mobile phases, A and B respectively. A constant flow rate (0.5 mL/min) was maintained throughout the gradient elution, which consisted the following sequence of solvent mixing: initially, phase B was set at 25% (1 min), then the concentration was changed to 95% over 10 min in a linear manner and maintained at this condition for 2 min. Column was re-equilibrated for 3 min after each injection. The column temperature was kept constant at 35 °C. Samples were stored at 4 °C throughout the analysis, and 10 μL of the sample was injected. The PDA detector was set at a range between 210 nm and 400 nm with 2-channel monitoring at 275 nm and 330 nm.

The tandem mass spectrometer consisted of an atmospheric pressure ionization (API) probe and for analysis of SP and CAN, positive ion mode (ES+) and for SA and SG, negative ion mode (ES−) were selected, with the condition tuned based on each authentic standard for identification purpose. The general MS/MS parameters were as follows: cone gas (N_2_) flow, 50 L/h; source temperature, 100 °C; desolvation temperature, 400 °C; capillary voltage, 3.00 kV; desolvation gas (N_2_) flow, 400 L/h; cone voltage, 25 V except for SP (22 V) and collision energy, 15 eV. The precursor to product ion transition was monitored using Multiple Reactions Monitoring (MRM) mode: SP, *m/z* 310 > 251; CL, *m/z* 181 > 121; SA, *m/z* 223 > 208 and SG, *m/z* 385 > 205. Daughter ion mode was used to obtain MS/MS spectrum of their precursor ions (also molecular ions except *m/z* 310 for SP). Mass resolution was set at maximum.

### 2.7. Data Expression and Analysis

Means and standard deviations were based on triplicate values. Data on phenolic content of mustard and canola fractions and their antioxidant activity were statistically interpreted using one factor ANOVA. For multiple comparisons, Tukey mean separation was followed using the Statistical Analysis System Program (SAS Institute, Carey, NC, USA), where in *p* ≤ 0.05 was fixed as level of significance.

## 3. Results and Discussion

The information on extraction and analysis of phenolics from canola and mustard was focused on either whole seed or extracted-oil or the meal. There is limited data on the comparative profile of phenolics of hulls and cotyledons of canola and mustard. Phenolics are known for its heterogeneous distribution in various fractions of oil seeds. There are very few studies available on the *in-situ* distribution of phenolics in different sections of an oilseed. Krygier *et al*. examined the distribution of phenolics in rapeseed hulls and de-hulled flour [32]. However, the results might not be very suitable for comparisons as the authors used an alkaline treatment method which might have affected the structural attribute of the original phenolics and thereby, its quantification. Similarly, Liu *et al*. investigated the distribution of soluble and insoluble phenolics in certain varieties of rapeseeds [33]. In the present study, we avoided destructive and harsh methods of phenolic extraction but rather followed solvent extractions at optimum conditions for better stability of phenolics. The various by-products of canola and mustard have been previously suggested as a potential substrate for phenolics and the results of our study further strengthen the understanding of phenolic distribution prior to the objective of value addition.

A large number of hydroxycinnamic acid derivatives, with varied structural attributes such as sinapoyl, caffeoyl, coumaroyl, hydroxyferulolyl and ferulolyl esters, are found in mustard greens [34]. The phenolic profile of mustard seeds is less complex, and most of the hydroxycinnamic acids except SADs were not reported. In the extracts of crude mustard seeds, SP was the principal phenolic, while the free SA was detected only in trace amount. Previously, SP, SG and free SA were reported in mustard meal [25]. Data’s on the phenolic profile and antioxidant activity of hulls and cotyledons of mustard and canola are scarce. Thus, the current study was conducted to investigate the antioxidant properties of SA and its derivatives present in different fractions of canola and mustard. Methanolic (70%) extracts of defatted fractions were analyzed for the total phenolic contents following two methods—the Folin-Ciocalteau assay, and HPLC profiling based on diode array detection.

### 3.1. Phenolic Profile of Extracts from Canola and Mustard Seed Fractions

Hydroxycinnamate conjugates are characteristic of brassicaceous plants [34,35,36]. The shikimate/phenylpropanoid pathway is responsible for the production of SA and its conversion to *O*-ester conjugates via a system of multiple enzymes [37]. From the taxonomic point of view, the seed-specific SP can be used as a biomarker to group the members of family Brassicaceae [35]. For the biosynthesis of phosphatidylcholine, SP (a choline ester) might work as a storage vehicle, whereas sinapoyl esters might include UV protection of plants [37]. In the present study, the distribution of these phenolics in various fractions of canola and mustard seeds was investigated. Previously, the efficiency of different solvents (70% v/v) such as methanol, iso-propanol and ethanol for canola phenolic extraction was evaluated following HPLC-DAD and found that the methanol was more efficient to obtain SADs [29]. The methanolic extracts from canola seed, in comparison to ethanol or iso-propanol extracts, had a higher total phenolic content, which was mainly contributed by its phenolics (SP, SG and SA).

The total phenolic content of canola samples were assessed using HPLC and Folin-Ciocalteu method (Table 1). The total phenolic contents (mg/g) were 10.60 (canola seeds), 4.50 (canola hulls), 16.89 (canola cotyledons), 10.31 (mustard seeds), 6.24 (mustard hulls), and 10.60 (mustard cotyledons) when analyzed by Folin-Ciocalteu method. However, the HPLC analysis showed relatively higher total phenolics values than Folin-Ciocalteu method, except in canola hulls and mustard hulls (3.57 and 5.67 mg/g respectively). The total phenolic contents with regards to the HPLC method (mg/g) were 14.06 (canola seeds), 20.20 (canola cotyledons), 11.12 (mustard seeds), and 11.45 (mustard cotyledons). Phenolic content estimated by both methods indicated that cotyledons are a richer source of phenolics than the hulls. The results are comparable with those of other researchers who reported a total phenolic content ranges from 10 to 18 mg/g [28,29,32,38]. Khattab *et al*. observed a total phenolic content (mg/g) of 17.71 (defatted canola seeds), 15.83 (canola meals) and 18.48 (canola seed press cakes) [29]. Kozlowska *et al*. emphasized that both the variety and processing methods affect the total phenolic content of canola meal (6.4–18.4 mg/g) [39]. Moreover, Cai and Arntfield indicated an insignificant difference between the total phenolics in the methanolic extracts of canola flour based on two estimation methods in which 22.90 and 22.58 mg/g were reported for Folin-Ciocalteu and HPLC methods respectively [23]. The varietal genetics, environment and the extent of maturation of seeds will determine the profile of phenolic constituents and thereby the content of total phenolics.

**Table 1:** Profile of sinapic acid and its derivatives in different fractions of mustard and canola with their EC_50_ values.

Samples	Sinapoyl Glucose	Sinapine	Sinapic Acid	Total Phenolics (HPLC) *	Total Phenolics Folin-Ciocalteu	EC_50_
Canola Cotyledon	8.71 ± 0.76 ^a^	12.20 ± 1.16 ^a^	0.22 ± 0.02 ^b^	20.20 ± 1.85 ^a^	16.89 ± 0.69 ^a^	1.78
Canola Seeds	5.45 ± 0.35 ^b^	8.35 ± 0.44 ^c^	0.15 ± 0.01 ^d^	14.06 ± 0.71 ^b^	10.60 ± 0.81 ^b^	2.31
Canola Hulls	1.34 ± 0.07 ^c^	1.15 ± 0.07 ^e^	0.04 ± 0.00 ^e^	3.57 ± 0.20 ^d^	4.50 ± 0.16 ^d^	5.82
Mustard Cotyledon	0.67 ± 0.01 ^c^	10.62 ± 0.08 ^b^	0.18 ± 0.00 ^c^	11.45 ± 0.05 ^c^	10.60 ± 0.24 ^b^	2.36
Mustard Seeds	0.66 ± 0.01 ^c^	10.17 ± 0.27 ^b^	0.19 ± 0.02 ^c^	11.12 ± 0.39 ^c^	10.31 ± 0.32 ^b^	2.54
Mustard Hulls	0.41 ± 0.02 ^c^	4.74 ± 0.28 ^d^	0.90 ± 0.01 ^a^	5.67 ± 0.32 ^d^	6.24 ± 0.40 ^c^	4.40

All the values (mg/g) except for EC_50_ are average and SD, while EC_50_ values are expressed in mg/mL, *n* = 3, values with different superscripts were significantly different at *p* *≤* 0.05. * Expressed as sinapic acid equivalents (SAE).

**Figure 1:**
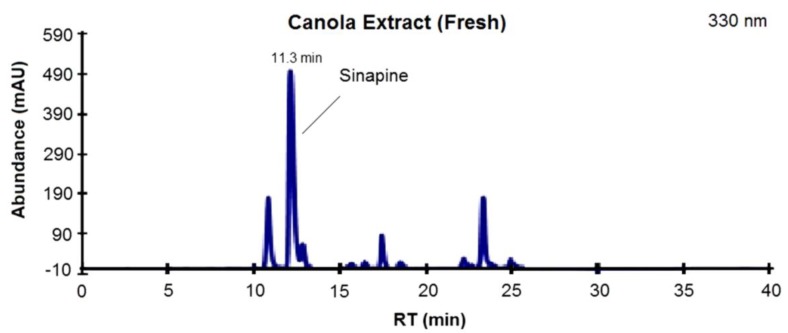
A representative high-performance liquid chromatography-diode array detector (HPLC-DAD) chromatogram (330 nm) of phenolic extracts from canola cotyledons showing the principal component sinapine (RT 11.3 min).

The HPLC-DAD was carried out at 330 nm (SADs) and 270 nm (CAN). The concentration of major SADs in canola and mustard extracts are shown in Table 1. Previously, Khattab *et al*. reported that SP solely represented about 69%–87% of the total phenolics of various canola fractions (seeds, meal and press cakes) [29]. A similar pattern was observed in the present study with respect to the canola variety analyzed (Nexera). The canola hulls had the lowest SP (1.15 mg/g) in comparison with canola seeds (8.35 mg/g), and canola cotyledon (12.20 mg/g). Interestingly, the concentration of SA was significantly less (0.04–0.22 mg/g) and this corresponds to 0.8%–1.09% of the total phenolics. Figure 1 represents a typical HPLC-DAD chromatogram (330 nm) of phenolic extracts from canola cotyledons showing the peaks of SG, SP and SA. It is well known that the rapeseeds have the highest amount of phenolics among various oilseeds of commercial origin, and such phenolics are leached into the oil during pressing the oil from rapeseed. Depending on the processing parameters, the amount of such phenolics in rapeseed oil will vary, for example, cold-pressed rapeseed oil and refined rapeseed oils contain 3–4 mg/kg and 16 mg/kg (caffeic acid equivalents) phenolics respectively [13,40]. The analysis of phenolics from Nexera canola variety indicated that the major phenolic was SP, with trace amounts of SA. Interestingly, the seeds and cotyledons of this canola variety had a significantly higher concentration of SG. Moreover, it was also found that the total phenolic concentration of cotyledon was relatively higher than other fractions of canola or mustard.

Recently, Siger *et al*. [41] identified and quantified SADs in the crude extracts of *Brassica napus* L. seeds as well as extracts after acidic and alkaline hydrolysis and indicated a high content of total phenolics (1577–1705 mg/100 g SA equivalent) in the crude extracts. 1-*O*-β-d-glucopyranosyl sinapate was the major SADs with the highest antioxidant capacity [41]. However, the concentrations of SG (4% of the total phenolics) as well as the total phenolics were much lower in *Brassica juncea* seeds in comparison to *Brassica napus*. Interestingly, SP and SA contents were insignificantly different. Both the mustard and canola cotyledons showed high content of oil than seeds or hulls (Table 2).

**Table 2:** Oil and moisture contents of seeds, hulls and cotyledons of mustard and canola.

Samples	Oil content % (Dry wt)	Moiture %
Canola Cotyledon	53.82 ± 0.66 ^a^	3.17 ± 0.00 ^f^
Canola Seeds	41.70 ± 0.62 ^b^	6.45 ± 0.09 ^c^
Mustard Seeds	38.35 ± 1.08 ^c^	5.10 ± 0.11 ^e^
Mustard Hulls	27.69 ± 0.07 ^d^	5.53 ± 0.13 ^d^
Canola Hulls	18.00 ± 0.09 ^e^	6.71 ± 0.04 ^b^
Mustard Cotyledon	42.18 ± 0.00 ^b^	7.06 ± 0.05 ^a^

All the values are average and SD, *n =* 3, values with different superscripts were significantly different at *p* ≤ 0.05.

In the case of mustard also, the major phenolic was SP, with the content of SP been significantly higher in both seeds and cotyledon in comparison to hulls (Table 1). In all of these fractions, both SG and SA were present only in minute quantities. Like canola hulls, the mustard hulls also showed the lowest SP (4.74 mg/g) in comparison with mustard seeds (10.17 mg/g) and mustard cotyledon (10.62 mg/g). There was no significant difference between the SP content of seeds and cotyledons. Interestingly, the concentration of SA was significantly higher (0.9 mg/g) in mustard hulls than mustard seed or cotyledon. The SG content of mustard seeds and cotyledons were almost same (0.66 and 0.67 mg/g respectively) and is higher than mustard hulls (0.41 mg/g). Between canola and mustard, the SG content was significantly higher in canola with a maximum of 8.71 mg/g in cotyledon followed by 5.45 and 1.34 mg/g in seeds and hulls respectively.

In our study, CAN was not detected in any of the samples, probably because CAN would be produced only through decarboxylation of SA during roasting of mustard seed and canola [13,17,18,42]. Since the CAN synthesis is majorly dependent on the partial hydrolysis of other esterified SADs while roasting, the relatively low content of free SA in the unroasted oilseed is not sufficient enough to produce it [42,43].

### 3.2. UPLC-MS Analysis of Phenolics from Mustard and Canola Cotyledons and Hulls

**Figure 2:**
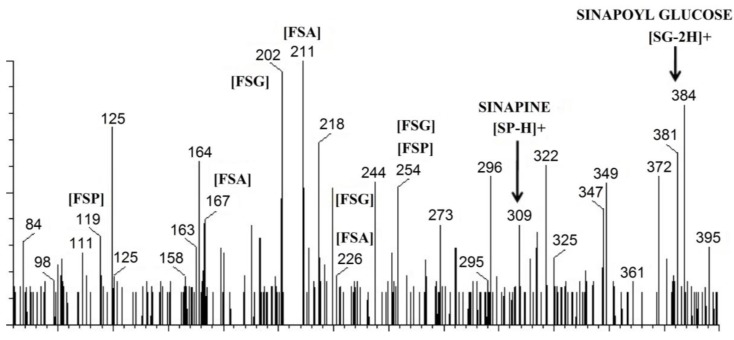
A typical ultra-high performance liquid chromatography-mass spectrometry (UPLC)-Mass spectrum of phenolics in the extracts of mustard cotyledons showing major fragments of sinapates. (SG: sinapoyl glucose, SP: sinapine, SA: sinapic acid, FSG: fragments of SG, FSP: fragments of SP, FSA: fragments of SA).

For UPLC-MS analysis, methanolic extracts of canola and mustard phenolics were used and compared with the fragmentation pattern of standard SP, SA and SG. Since the type of molecules in the phenolic extracts of hulls and cotyledons of canola and mustard and their basic fragmentation pattern are similar, only a typical UPLC-MS of phenolic extracts obtained from mustard cotyledons is presented (Figure 2). SP was the predominant substance in the polyphenolic fractions of seed, cotyledons, and hulls of canola as well as mustard as indicated by HPLC-DAD. Based on UPLC-MS data, ions at *m/z* 254, and its breakdown product at *m/z* 119 tentatively identified SP as the choline ester of SA (Figure 2). While SP is generally regarded as the major phenolic compound in most of the brassicaceous species, data on the fragmentation pattern using mass spectrometry is scarce. The characteristic major (*m/z* 251) and minor (*m/z* 207, 175, 147, 119 and 91) fragments of standard SP were observed (spectrum not shown). The fragmentation pattern noticed in the present investigation was comparable with earlier observations for phenolics obtained from extracts of canola seed [44,45]. Even though, the signal at *m/z* 309, which corresponded to the [M – H]^−^ ion was observed, a signal at *m/z* 311 ([M + H]^+^) ion was absent. With respect to the ionization of phenolic compounds from mustard and canola fractions, our study indicated that a positive mode is best suited for detecting SP (*m/z* 310) in its molecular ion form.

SA in the canola and mustard fractions was readily identified based on MS spectra, UV data, and comparison with respective reference standard. Hydroxycinnamic acids produce two fragments (*m/z* 208 and 193) by losing two methyl groups and are characteristics of hydroxycinnamic acid fragmentation. The standard SA exhibited its characteristic fragmentations at *m/z* 223 and 164 (spectrum not shown). The major fragments of SA were found at *m/z* 223, 208, 179, 164 and 149 while the minor fragments were observed at *m/z* 193 and 147. In the present study, when the canola and mustard phenolics were analyzed, some of these fragments were detected at *m/z* 226, 211 and 167, corresponding to [M + 3H]^+^ ions of fragments of SA standard. Sinapate esters, which showed the similar fragmentation pattern, were also characterized. The MS analysis of the current study matched with the results by Engels* et al*. [45].

Presence of [M – 2H]^+^ ion at m /z 384 confirmed the presence of sinapoyl hexose, possibly SG (Figure 2). Typical fragmentation pattern of SG consists of fragments at *m/z* 251, 223, 205, 190, 179 and 164. When mustard and canola phenolics were analyzed, two of these fragments (*m/z* 254 and 226) corresponding to [M + 3H]^+^ ions of fragments and two signals (*m/z* 384 for the parent molecule and 202) corresponding to [M – 3H]^+^ ions of fragments of standard SG were observed.

### 3.3. DPPH Scavenging Effects of Various Phenolic Extracts

Among various methods used to evaluate the antioxidant activities of plant phenolics, the DPPH radical assay is very common and reliable [46,47,48]. The ratio of reduction in absorbance (517 nm) of DPPH solution in the presence and absence of phenolics is widely used as an estimate for radical-scavenging activity of an antioxidant [47]. This procedure was later modified to consider various kinetic properties of antioxidants [46]: however, the modification was not apt to assess the antioxidant potential of phenolic extracts (crude), because the idea of structural characteristics of molecules is imperative. In the present study, to overcome this limitation, EC_50_ was suggested as an appropriate expression of radical-scavenging potential. EC_50_ was defined as the quantity of phenolic extract (crude) needed for a half decrease in the concentration of DPPH radicals during assay [31]. A low EC_50_ value corresponds to a strong radical-scavenging activity.

**Figure 3:**
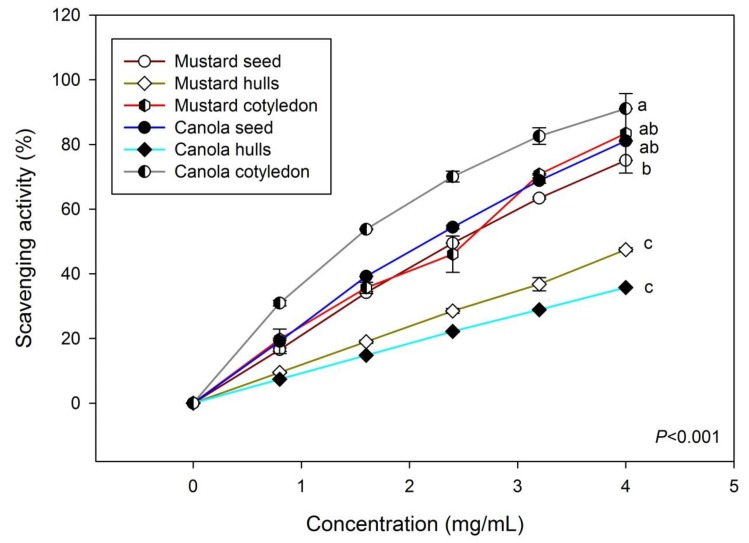
1,1-diphenyl-2-picrylhydrazyl (DPPH) scavenging activity (%) of phenolic extracts from seeds, cotyledons and hulls of Nexera canola and mustard. (Values with different letters were significantly different at *p* < 0.001).

Previously, Khattab *et al*. investigated the antioxidative efficacy of minor components (phytic acid, chlorophyll, and condensed tannin) of a few varieties of canola (*Brassica napus* L.) seeds, meals and cakes in comparison to one Indian mustard (*Brassica juncea* L.) [49]. In this study, the authors did not focus on the scavenging effects of SADs. In our study, we observed that phenolic extracts of canola cotyledon is significantly more potent than other extracts in terms of its effectiveness to scavenge DPPH (Figure 3). Among the various phenolic extracts from mustard, the cotyledon extract showed more scavenging activity than extracts of whole seed or hulls while the scavenging efficacy of mustard hull phenolics is comparatively lower than others. The result also indicated that the EC_50_ concentration (mg/mL) of these phenolics followed a decreasing order of scavenging activity: canola cotyledon > canola seed > mustard cotyledon > mustard seed > mustard hulls > canola hulls (Table 1). A high degree of correlation was established between DPPH scavenging activity (%) and total phenolic content (*r* = 0.93). Utilization of canola hulls as a potential substrate for anti-oxidants extraction was discussed previously [50]; however, there were no comparative data for hulls and cotyledons in comparisons with canola and mustard. The present study is relevant in this perspective.

## 4. Conclusions

Aqueous methanol (70%) was the best extraction solvent to recover phenolics, especially SADs from canola and mustard seeds and their fractions (hulls and cotyledon). The major phenolic compound in both hull and cotyledon extracts was SP with a relatively smaller amount of SA and SG, with significant variation between the two seed fractions. Canolol was not found in the crude extracts investigated in this study. Further, the DPPH scavenging activities of the methanolic extracts indicated a co-relation with their total phenolic content. Based on the quantitative profile of SADs, the major contributor towards the antioxidant potential is SP. Even though UPLC-MS confirmed the presence of SG, SP and SA in the extracts, detailed studies on their fragmentation pattern are needed to establish the structural changes and formation of novel antioxidants like canolol from SADs. In order to translate the results of this study to food development and applications, for example, the effects of various phenolic fractions of canola and mustard seeds in bulk oil and emulsion systems, more research is warranted. The study also implies that most of the phenolics are accumulated in cotyledons of canola and mustard and only a fraction of it is found in hulls. This information on the quantitative distribution of SADs is certainly a catalyst to add value to by-products for recovery of endogenous phenolics either to add back to oils or to fortify other lipid-systems.
